# Who Gets Dental Caries? A Comprehensive Review

**DOI:** 10.3390/dj14070400

**Published:** 2026-07-02

**Authors:** Svante Twetman, William Papaioannou, Sotiria Gizani

**Affiliations:** 1 Department of Odontology, Faculty of Health and Medical Sciences, University of Copenhagen, DK-2200 Copenhagen, Denmark; 2 Department of Paediatric Dentistry, School of Dentistry, National and Kapodistrian University of Athens, 11527 Athens, Greece; stgizani@dent.uoa.gr; 3 Department of Preventive and Community Dentistry, School of Dentistry, National and Kapodistrian University of Athens, 11527 Athens, Greece; vpapaio@dent.uoa.gr

**Keywords:** caries trajectory, children, heredity, risk assessment, socio-economy, sugars

## Abstract

Dental caries is the world’s most common non-communicable disease with a complex etiology including social, behavioral, medical, biological and economical elements. The distribution among populations and age groups is skewed, which calls for validated clinical tools to identify those with increased caries risk. The aim of this article was therefore to review risk factors for caries development in children, based on global prospective birth cohorts, genetic proceedings and data from validated risk assessment tools. The genetic elements involve four main categories comprising enamel quality, salivary composition, dental biofilm function and taste preferences. Recent studies suggest that genes may account for 35–55% of the variation in caries scores in the young permanent dentition. Prospective birth cohorts have pointed out poverty, socioeconomic level, early introduction and excessive sugar intake as significant factors for a child’s dental caries trajectory up into adulthood. Moreover, prolonged breastfeeding, child obesity and maternal oral conditions are linked to the caries burden later in life. In the clinic, the strongest predictors are past caries history and selected behavioral and social factors. The performance of the validated caries risk assessment models is far from perfect, but still acceptable in terms of reliability during childhood. These tools are the best clinical practice since they add objectivity, consistency and documentation to the clinical routines. In addition, the dental staff has the advantage of using the outcome of the assessment for a structured risk communication with the caregivers. The protocol should also form the basis for a personalized intervention program addressing the entire family, with particular focus on maternal oral health and sugar reduction.

## 1. Introduction

Dental caries is a common non-communicable disease that affects over 500 million children worldwide [1] and if left untreated, the condition is associated with a reduced quality of life in terms of pain, infections, eating problems, learning difficulties, and malocclusions [2]. Caries is described as a preventable disease, yet the incidence has increased over the past decades and is projected to continue to increase with the growth of global population [3]. Another concern is that the burden of caries is unevenly distributed with large variations across countries, regions, cities, as well as between populations of different cultural backgrounds, socioeconomic status and education level [1]. A common expression is that “20% of the population has 80% of the disease” [4] and this highlights the challenge to identify children with caries risk early in life. Focusing on those with the highest risk and need is a proven strategy to reduce future inequalities in oral health [5]. Furthermore, caries in childhood is linked to metabolic and cardio-vascular diseases in adult life [3,6,7,8], as well as poorer mental health and well-being [9]. Consequently, caries risk assessment (CRA) followed by a personalized care plan is an obvious cornerstone in the prevention and management of dental caries [10]. There are several comprehensive CRA protocols available for children that include past caries experience and a range of biological, behavior and social background factors, but such tools have not widely entered clinical practice. Practice-based research indicates that a minority of dentists (10–30%) use a structured form, or tool, to assess caries risk for children and adults, leading to a subsequent gap in the individual care plan [11,12,13,14]. Most dental professionals seem to rely on an intuitive assessment (“gut feeling”) based on personal experience rather than education and formal clinical training [11]. The pertinent question is therefore “which child will get caries?” According to a systematic review, there are over 100 risk factors significantly related to the prevalence or incidence of dental caries in children, albeit most of them obtained from cross-sectional studies [15]. All these risk factors may very well be relevant and important, but in order to establish true causality, prospective trials are required. Evidence of highest certainty comes from prospective birth cohorts. In addition, genetic studies in recent years have provided information on individual predisposition and vulnerability for caries. There are indeed separate papers available on genetics, caries risk factors, caries trajectories and risk assessment protocols but as far as we know, a broad overview for clinicians and researchers, covering these aspects, seems to be lacking. The aim of this article was therefore to review and communicate the risk factors for caries development in children based on global birth cohorts, genetic proceedings and data from validated risk assessment tools.

## 2. Search Strategy

We based this paper on systematic and comprehensive reviews published in English. We searched two separate databases (PubMed and The Cochrane Library) for the relevant literature from 1 January 2010 up to 1 March 2026. We used the following main search words: “dental caries/tooth decay “infants/children/adolescents”, “prediction”, “prognosis”, “longitudinal study”, “birth cohort”, “candidate gene”, “genetic variation” and “nucleotide polymorphisms” in different combinations, as detailed in Supplementary File Tables S1 and S2. Initially, 858 review papers were retrieved. Two authors (ST and SG) screened the abstracts independently for relevance and the final inclusion of 24 review papers was based on consensus. In addition, we included selected primary studies of particular interest to the discussion. We did not perform a formal risk of bias assessment of the included papers.

## 3. Prospective Birth Cohort Studies

Population-based birth cohort studies have the most appropriate design to determine the causal relationship between potential risk factors and the health status from newborn up to childhood and potentially adulthood [16]. This means that data are collected from individuals born during a certain timeframe and at various intervals during growth. In addition, maternal data collected during pregnancy can be included and analyzed. However, the relatively large size of most birth cohorts means that results are calculated and reported on group level rather than on individuals, which is somewhat limiting. One of the first and well-known studies of this kind is the Dunedin Multidisciplinary Health and Development Study, which has now been ongoing for half a century [17]. Today, there are over 100 ongoing birth cohorts from 34 countries on all continents that include oral health [18] and findings from 15 of the major studies have been summarized by Peres and co-authors [19]. Collectively, they show that poverty and low socioeconomic circumstances during childhood have a long-lasting negative influence on the caries levels up to adult ages. They also found a clear co-variation between the socioeconomic gradient and early sugar introduction and excess energy consumption (>5% from free sugars). Moreover, a regular and frequent intake of sugar from various sources early in life increased the caries experience in adolescence and adulthood [19]. Prolonged breastfeeding (after 11 months of age) was closely associated with early childhood caries. Access to care was another important factor since long-term unfavorable patterns of dental visits were associated with poor adult health. Among general health factors, early-life obesity was associated with increased levels of dental caries among adolescents [19]. Another outstanding factor was that the maternal oral conditions were predictive of the child’s caries development and its oral health-related quality of life in adulthood [19]. Thus, available birth cohorts underline the importance of documenting the parents’ socioeconomic and oral health status and registering this information into the dental records together with the timing of sugar introduction and dental attendance, as this may assist the dental staff to deliver personalized information to prevent and manage dental caries in childhood.

## 4. Genetic Components

It is a common saying among patients that “I have inherited my parents’ bad teeth”, but the genetic component of dental caries has traditionally gained relatively little attention. Already the infamous Vipeholm study in Sweden back in the 1950s reported an individual susceptibility to caries, as 40% of the participants with a very frequent daily intake of sticky toffees did not develop any new caries lesion over a 2-year period [20]. In recent years, genome-wide association studies have linked various genes to functions that may make teeth more vulnerable to dental caries. They can be grouped into four slightly overlapping categories, namely those affecting enamel quality, salivary composition, biofilm function or taste preferences. We show some examples in Table 1. The genes that have a direct impact on the caries process are those involved in the tooth formation and the quality of enamel [21], while those that affect the salivary composition, dental biofilm diversity and taste preferences exert a more indirect influence on the disease [22]. It is difficult to estimate how much of the caries disease can be explained by a genetic predisposition since the expression of selected genes can be modulated by epigenetic and environmental factors [23]. However, a study has suggested that genes may account for 54–70% of the variation in caries scores in the primary dentition and 35–55% in the permanent dentition [24]. The higher impact in preschool children may be explained by a more susceptible enamel in primary teeth but also by factors related to the development of the oral microbiome that is most dynamic during the first 1000 days of life [25,26]. In addition, individual taste preferences are likely to play a significant role in the development of early eating habits. The interaction between genetic and environmental determinants remains also to be further explored. For example, it is likely that the susceptibility to caries is linked and combined with environmental triggers such as diet and lifestyle choices. Today, genetic tests for dental caries that may identify DNA variations for enamel quality and saliva composition through saliva swabs are available but under validation for children’s caries risk [27]. Such convenient tests will most likely appear in the future. Meanwhile, the parents’ (and siblings’) oral conditions may give a hint on the future dental health of the child.

## 5. Accuracy of Caries Risk Assessment (CRA) Tools

The primary goal of CRA is to identify individuals with increased risk of caries and this includes both the risk for new lesions and the progression of already existing lesions. The strength of the clinical evaluations of various risk assessment models is that they provide data on the individual level. The research on caries prediction is, however, associated with ethical dilemmas. The accuracy of the risk assessment tools or models must be established in double-blind randomized prospective trials (index test vs. reference test), and it is not possible to include a control group that receives less than the “best available standard care” or “treatment as usual”. Therefore, the predictive values from different populations may be flawed and biased, in particular when the standard preventive care is effective. In addition, CRA tools validated in different countries and populations with different socioeconomic standards and dental care systems may suffer from a low external validity. Consequently, the performance of the tools should generally be interpreted with caution. Data from systematic reviews suggest that the performance of available caries risk models are far from perfect, but still acceptable in terms of reliability during childhood with a reported area under the curve (AUC) values ranging from 0.57 to 0.91 [28,29]. A perhaps more understandable measure is that a child with an elevated caries risk (Positive test) has 5–10 times higher chance to develop caries in the future (positive likelihood ratio > 5) [30]. The single best predictor for new caries lesions or progression of existing lesions is the past caries experience increment or simply past caries [31] and this is understandable in the light of prospective studies that have shown that caries increment shows a constant trajectory throughout life [32]. Other predictors of importance identified in systematic reviews are (i) behavioral factors (snacking and feeding habits, oral hygiene behavior, fluoride exposure, and “dental home” attendance), (ii) sociodemographic factors (child’s age and family status, poverty), (iii) clinical factors such as level of oral hygiene, and (iv) parents’ oral health [33,34]. Systematic reviews show, however, that a combination of predictors (three or more) is more useful and accurate than single predictors [30,33]. In Table 2, we provide a list of some of the caries risk factors that should be included in a comprehensive CRA process in children. Moreover, reference manuals and recommendations for risk assessment in infants, children, and adolescents are available from international pediatric dentistry organizations [35,36].

In previous years, several CRA tools included a measure of salivary bacterial counts (e.g., mutans streptococci) but simple chair-side tests are no longer commercially available in most countries. This is no major drawback since the selective bacterial counts, in general, display a high specificity but a low sensitivity for caries lesion development [31]. Today, high-throughput sequencing platforms are available to analyze the complete oral microbiota, and this may change the scene. A systematic review has compiled recent data from 13 such studies [37] and found the sensitivity to range from 0.6 to 1.0 and the specificity to be between 0.75 and 0.90. The included studies had a high risk of bias and the authors concluded that the available evidence did not support the use of microbial composition as a screening test to identify children at risk for caries development. Another interesting area of rapid progress is artificial intelligence (AI) based on machine learning and deep learning that can aid dental practitioners in caries prediction and clinical decision-making in children. Systematic reviews have demonstrated a good diagnostic performance with a pooled sensitivity and specificity over 0.8 [38,39]. Thus, the predictive accuracy of AI seems to well match or exceed that of experienced dentists. Interestingly, the deep learning architectures consistently outperformed traditional machine learning approaches [39]. However, further external validation and real-world studies are needed for broader clinical implementation. In the near future, it is likely that the AI’s ability to predict will improve with the addition of more clinical data and genetic markers.

The today’s literature suggests that CRA models for prediction of caries increment in childhood are reliable enough in clinical practice for general populations. Thus, the use of any comprehensive CRA tool is best practice since it adds objectivity and consistency to the process and facilitates documentation in the dental charts. In addition, practitioners can take advantage of the outcome for structured risk communication with caregivers and offspring. For this purpose, the dental professional needs to adopt and incorporate behavior change theories into the caries-preventive toolbox [40]. The outcome of the caries risk protocol should also form the basis of a personalized intervention program addressing the entire family, with particular focus on maternal oral health and family sugar habits.

## 6. Discussion

Data from the global birth cohorts demonstrated unanimously the very close connection between poverty and low socioeconomic conditions and caries in early childhood. While social inequality requires structural policy changes and economic reforms, the co-variation with excess sugar intake can and must be addressed by dental professionals. The objective is not only to reduce the risk for early caries development but also to reduce the future risk of overweight and Type 2 diabetes and hypertension [41]. The World Health Organization and aligned international health authorities have recommended that infants and children under 2 years should have zero added sugars in their diet [42]. After that, free sugars in food, snacks and drinks should be less than 10% of the daily total energy intake, and the closer to 5% the better [42]. In this context, dental professionals can offer culturally tailored sugar reduction and behavioral support in cooperation with other health professionals, community centers and nursery schools.

Microbial analyses of dental biofilm samples will probably be more relevant than saliva samples in preschool children, reflecting the important first 1000 days of life. This is a critical window for oral microbiota development, directly influencing a child’s long-term dental and systemic health [25,26]. The detection of cross-kingdom communities seems of particular importance since birth cohorts suggest pre-schoolchildren harboring *Candida* spp. may have 4.5 times higher caries risk compared to those without these microorganisms in the oral cavity [43,44]. The non-invasive tests involving saliva swabs are easy to collect but the analyses and interpretation come with substantial costs. In addition, dental professionals must be aware of the moral and ethical questions associated with both genetic tests and the CRA tools. As caries is a multifactorial disease, a genetic test may not accurately predict when or if a child will develop cavities and this may result in unnecessary anxiety, social stigma and individual discrimination [21]. Obviously, sensitive genetic information data must be kept in a secure way in the dental records. The various CRA tools also have their concerns and limitations. Low validity implies that some children with increased caries risk will remain unidentified, whereas some can be falsely identified as being at risk. A general trend is that CRA tools tend to overestimate the caries risk and this may drive overtreatment and increased costs, both for the parents and for the society [45]. Furthermore, misclassifications can lead to stigma and suboptimal care; false “positive” tests to overtreatment and unnecessary fillings while a false negative outcome may be associated with neglected preventive care [46]. A final comment is that “risk” is a subjective value word that is hard to define and sometimes to understand [47]. Therefore, the risk communication with parents and the child should focus on the message and benefits of remaining “caries free”, as well as on the future lifelong consequences on oral and general health of having childhood caries.

We based this article on systematic and comprehensive reviews published in English covering the topic. Thereby, important information from primary studies, and studies published in other languages, may have been overlooked.

## 7. Conclusions

Dental caries is the world’s most common non-communicable disease, but the distribution is uneven among the population and age groups. The risk of developing caries in childhood is significantly related to caries and the presence of several systemic diseases in adulthood. The etiology is complex with social, behavioral, medical, biological and economical elements, and the crucial question “who gets caries?” is as complex to answer. Genome-wide studies indicate a substantial hereditary component, in particular influencing the enamel quality, and prospective birth cohorts have pointed out the families’ socioeconomic level, early excessive sugar intake and the maternal oral conditions are the major predictors of the child’s dental caries trajectory up into adulthood. The strongest predictors in validated CRA tools are the past caries history and selected behavioral and social factors. A comprehensive CRA tool is best practice since it adds objectivity, consistency and documentation to the clinical process. In addition, the dental staff has the advantage to use the outcome for structured risk communication with the caregivers. The protocol should also form the basis for a personalized intervention program, addressing the entire family with particular focus on maternal oral health and sugar reduction.

## 8. Future Directions

Early identification of children at caries risk is a key challenge to reduce inequities in oral and general health through adolescence and adulthood. Artificial intelligence plays a future role when developed to assist dental professionals in finding those with elevated caries risk by combining genetic, social and clinical data. This will likely improve the accuracy of the CRA process, but the challenge is, like in all caries trials, to validate the performance in relevant and appropriate study populations and to allow an adequate long-term duration (2–3 years). In this context, convenient and affordable genetic tests together with micro-swabs for dental biofilm sampling will pave the way to assess the child’s individual predisposition for future caries development and help dentists to create personalized preventive strategies for those with high risk.

## Figures and Tables

**Table 1:** Examples of genes making teeth more susceptible to dental caries. From Slobodnikova et al. 2024 [22].

Genes	Function	Role in Dental Caries
ENAM, AMLEX, TUFT1, MMP16, MMP20	Enamel structure and mineralization	Variations can lead to thinner, weaker and less mineralized and resistant enamel
CA6, DEFB1, LTF, MUC7	Salivary composition	Reduced calcium and phosphate levels; lower pH, buffering capacity and remineralizing potential of saliva. Low levels of enzymes and antibacterial proteins hamper the defense against caries-associated microorganisms
PRH1, PRH2, DRB1, MBL	Biofilm growth, composition and function; immune response	The individual immune response affects the early establishment and development of the oral microbiome. Regulate the proportion of saccharolytic bacteria in the dental biofilm
TAS1R2, TAS2R	Taste preference (sweet, bitter)	Variations in taste receptors can influence a preference for sweet foods, indirectly increasing risk

**Table 2:** Example of significant caries risk factors in children derived from prospective birth cohorts and genetic studies.

Caries Risk Factors	Explanation
Hereditary factors	
Parents and/or siblings have caries problems, unsound teeth	May indicate susceptible dental enamel/dentin, hypomineralized teeth, enamel defects, complex deep fissures Maternal oral conditions related to child’s caries risk
The child has previous caries activity	Caries trajectory up to adulthood
The child has sweet taste preferences	May indicate early introduction and excess intake of free sugars
General health	
Childhood obesity	Increased risk of caries in adolescence
Social factors	
Low childhood socioeconomic circumstances, poverty	Long-lasting negative influence on caries levels
Unfavorable pattern of dental visits, limited access to dental care	Linked to poor oral health in adulthood
Inability to comply, low motivation, parents smoking	Linked to poor oral health literacy
Clinical factors	
Poor oral hygiene with thick, visible plaque; gingivitis	Indicate non-regular or inadequate tooth brushing habits
Suboptimal daily exposure to fluoride	Associated with higher caries levels
Nutrition and dietary factors	
High/frequent intake of free sugars (sugary diet, snacking, sweet beverages, juice)	Linked to increased caries activity and frequency
Nighttime feeding (breast, baby bottles)	Prolonged breastfeeding (after 11 months) associated with early childhood caries, in particular when combined with suboptimal oral hygiene and fluoride exposure

## Data Availability

No new data were created or analyzed in this study. Data sharing is not applicable to this article.

## Supplementary Materials

The following supporting information can be downloaded at https://www.mdpi.com/article/10.3390/dj14070400/s1, Table S1: PubMed search for systematic reviews up to 1 March 2026. Table S2. Cochrane via Wiley (Cochrane Database of Systematic Review) up to 1 March 2026.
