# Supplementary material for: Who Gets Dental Caries? A Comprehensive Review
Source: Dent J (Basel). 2026 Jul 2;14(7):400. doi: 10.3390/dj14070400 (PMC13407877; doi:10.3390/dj14070400)
Supplement: Supplementary file 1 [file dentistry-14-00400-s001.zip › dentistry-4328555-supplementary.pdf]

Table S1. PubMed search for systematic reviews up to March 1, 2026

1	dental caries [Mesh] OR tooth decay, ti,ab,kw
2	infant OR child OR childhood OR adolescence; ti,ab
3	dental caries susceptibility/genetics [Mesh] OR candidate gene OR genetic variation OR polymorphisms, single nucleotide [Mesh]; ti,ab,kw
4	longitudinal studies [Majr] OR birth cohort studies [Majr]
5	prediction [Mesh] OR prognosis [Mesh] OR risk assessment [Title/Abstract] OR forecast OR decision making
6	systematic review OR comprehensive review OR scooping review; ti
7	1 AND 2 AND 3 AND 6
8	1 AND 2 AND 4 AND 6
9	1 AND 2 AND 5 AND 6
10	7-9: limit language (English)
11	7-9: January 1 2010 to current

Table S2. Cochrane via Wiley (Cochrane Database of Systematic Review) up to March 1 2026

1	MeSH descriptor: [Dental Caries], ti,ab,kw
2	MeSH descriptor: [Dental Caries Susceptibility] OR genetics OR candidate gene OR genetic variation OR polymorphisms, single nucleotide, ti,ab,kw
3	MeSH descriptor: [Prediction] OR prognosis [Mesh] OR risk assessment, ti,ab,kw
4	MeSH descriptor: [Birth cohorts] OR cohort study ,ti,ab,kw
5	Limit to Jan 1 2010
6	1 AND 2 AND 5; 1 AND 3 AND 5; 1 AND 5 AND 5

ti = Title; ab = Abstract; kw = Keyword
